# Case report: IORT as an alternative treatment option for breast cancer patients with difficulty staying still

**DOI:** 10.3389/fonc.2024.1429326

**Published:** 2024-09-24

**Authors:** Fardeen Bhimani, Maureen McEvoy, Yu Chen, Anjuli Gupta, Jessica Pastoriza, Shani Fruchter, Zachary C. Bitan, Wolfgang A. Tomé, Keyur Mehta, Jana Fox, Sheldon Feldman

**Affiliations:** ^1^ Breast Surgery Division, Department of Surgery, Montefiore Medical Center, Montefiore Einstein Comprehensive Cancer Center, Bronx, NY, United States; ^2^ Department of Radiation Oncology, Montefiore Medical Center, Montefiore Einstein Comprehensive Cancer Center, Bronx, NY, United States

**Keywords:** breast cancer, intraoperative radiotherapy, TARGIT, IORT, intellectual disability, schizophrenia, bipolar disorder, psychiatric disorders

## Abstract

**Background:**

Administering radiation therapy to individuals with intellectual disabilities (ID) and psychiatric patients taking antipsychotics poses challenges, especially with whole breast irradiation (WBI) due to difficulty staying still (DSS). In such scenarios, intraoperative radiotherapy (TARGIT-IORT) provides an alternative. Although prior studies have shown its applicability in special cases where WBI may be contraindicated, there is a paucity of literature emphasizing its role in patients with ID and psychiatric conditions who have DSS. Therefore, our case series aims to highlight the applicability of administering TARGIT-IORT in such patients.

**Case reports:**

Four breast cancer patients underwent lumpectomy and TARGIT-IORT. Among them, two patients had ID, with one experiencing a decreased range of motion. The other two had psychiatric disorders, including schizophrenia and bipolar disorder, both manifesting involuntary movements and DSS. Three patients had invasive ductal carcinoma (IDC), and one had invasive lobular carcinoma (ILC). All patients undergoing TARGIT-IORT tolerated the procedure well. Notably, none of the patients exhibited evidence of disease on follow-up.

**Conclusion:**

Our study underscores the potential use of TARGIT-IORT as a viable treatment option for breast cancer patients with intellectual and psychiatric disabilities. Unlike traditional EBRT, TARGIT-IORT offers a single radiation dose, addressing challenges associated with compliance or DSS. Our findings demonstrate positive outcomes and tolerance, especially in patients where standard oncologic procedures are difficult to achieve. TARGIT-IORT could also benefit breast cancer patients with concurrent movement disorders like Parkinson’s disease and other movement disorders. Nonetheless, future studies are needed to reinforce its applicability for patients with DSS.

## Introduction

Breast cancer is the most common newly diagnosed malignancy among women across the United States (1). Of all the cancers diagnosed in women, breast cancer accounts for about 30% of the cases (2). It was estimated that approximately 310,720 women will be diagnosed with breast cancer in 2024 (3). Historically, mastectomy was considered the sole treatment option, even for early-stage breast cancer. However, numerous studies have indicated that adopting a more conservative surgical approach together with adjuvant radiation for small breast cancers can yield comparable long-term outcomes in locoregional recurrence and survival. This shift has led to a transition from mastectomy to breast-conserving surgery (BCS) involving quadrantectomy and, eventually, lumpectomy for early breast cancer (4–6). Similarly, a change in preference has also been seen in selected patients undergoing radiation therapy, with patients receiving partial breast irradiation (PBI) delivered over 1-3 weeks instead of the traditional whole-breast irradiation (WBI). Another option is intraoperative radiotherapy (TARGIT-IORT), which allows for a single dose to be given at the time of lumpectomy. The appeal of TARGIT-IORT lies in its intrinsic advantages of tissue preservation, as well as convenience, making it a popular choice among patients (7–10).

The administration of daily external beam radiation requires patients to lie flat and still, and to abduct the ipsilateral arm above the level of the shoulder. This can be a significant challenge for patients with intellectual disability (ID) and psychiatric disorders (11, 12). Individuals with ID often find it difficult to remain still, and psychiatric patients on antipsychotics may experience bradykinesia, akathisia, and tardive dyskinesia (13). This can make radiation treatments potentially unsafe or even impossible, and thus poses a potential contraindication for radiation in patients with difficulty staying still (DSS). While the occurrence of breast cancer among women with ID is comparable to that in the general population (14–16), studies have revealed a significantly increased risk of overall and breast cancer-specific mortality in this group (17, 18). Similarly, psychiatric patients have a similar incidence of breast cancer as the general population but face higher mortality rates (19). The rising incidence of breast cancer, coupled with a growing population of individuals diagnosed with ID (14, 20–22) as well as those with psychiatric disorders who frequently experience delayed diagnoses of breast cancer (23, 24), may present a potential threat to overall breast cancer survival rates. In such scenarios for patients with DSS, TARGIT-IORT presents itself as an attractive radiation alternative that allows for a single dose to be administered at the time of BCS. Prior studies have documented that TARGIT-IORT can be highly advantageous in specific scenarios, such as for patients with prior breast cancer seeking a second chance at breast conservation, those with movement disorders like Huntington’s or Parkinson’s, multiple sclerosis, wheelchair-bound, autism, or other patients with DSS, those struggling with radiation cycle compliance, or individuals who have previously received mantle radiation (25–29). In our study, we defined DSS as any patient with neurological, psychiatric, and/or developmental problems resulting in movement disorder, thereby impeding their capacity to undergo external beam radiation (EBRT). Although prior studies have shown TARGIT-IORT’s applicability in special clinical cases selected as per the ASTRO criteria where WBI might be contraindicated (30–33), there is a paucity of literature examining the role of TARGIT-IORT in patients with ID and psychiatric illness with DSS. Thus, our case series aims to highlight the applicability of administering TARGIT-IORT in such patients.

## Case reports

### Patient 1

A 66-year-old woman with severe developmental delay and behavioral disorder, who is non-verbal and unable to perform range of motion due to cognitive limitations, underwent a routine bilateral screening mammogram and ultrasound in 2014, which revealed a 1.3 cm mass in the 2:00 axis in the left breast and a 0.3 cm simple cyst in the right 7:00 axis. A follow-up mammogram revealed a 2 cm mass in the left lateral breast, and the ultrasound showed a 1.7 x 1.3 x 1.5 cm mass at the left 1:00, 4 cm from the nipple. From 2014 to 2018, successive annual ultrasounds documented a reduction in the size of the left breast mass, progressively diminishing from 1.8 cm to 1.2 cm to 0.7 cm to 0.4 cm, eventually disappearing completely. However, a follow-up ultrasound in 2019 detected a new 0.8 cm mass in the right breast at the 1:00 axis. The patient was unable to cooperate with an ultrasound-guided biopsy for a suspicious lesion in the right breast. Given her condition, she was deemed unfit for surgery and advised to undergo regular follow-up ultrasounds. A targeted ultrasound performed 9 months later demonstrated the right breast mass increased to approximately 1.1 to 1.5 cm from its previous size. Left breast ultrasound showed no mass, which was previously present. A biopsy of the right breast was eventually performed in the clinic, and it revealed grade 3 poorly differentiated invasive ductal carcinoma (IDC). The tumor was diagnosed as clinical stage IA T1 N0 M0, and tumor markers were ER-positive, PR-positive, and Her-2 positive by FISH (signal ratio of 2.11 and a copy number of 7.0.) The patient’s case was discussed in the Multidisciplinary Tumor Board (MTB), and it was collectively decided to offer neoadjuvant treatment. Following this, she started a combination of subcutaneous pertuzumab/trastuzumab/hyaluronidase. Upon completion of this course, she was started on exemestane. The patient’s case was again discussed in MTB, and it was collectively decided to treat her with curative intent and offer her lumpectomy with sentinel lymph node biopsy and TARGIT-IORT.

**Figure 1:**
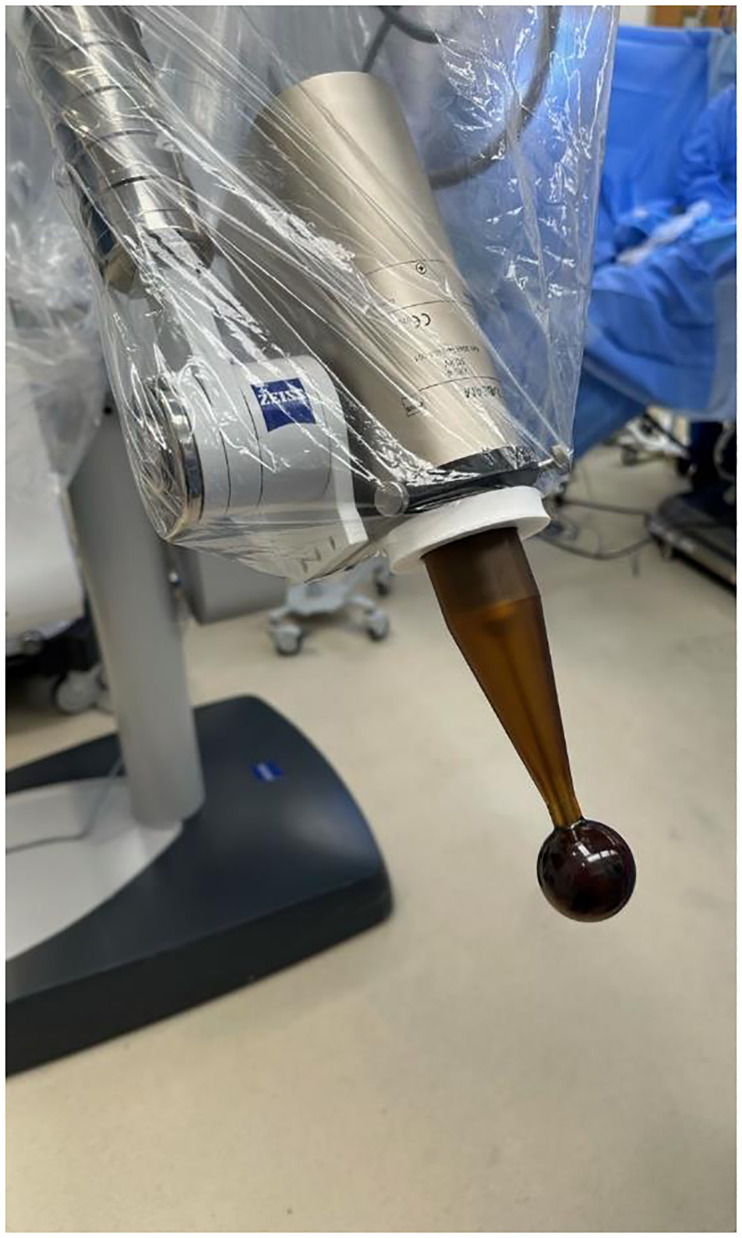
Dressed Intrabeam 600 miniaturized 50 KV X-Ray linear accelerator with 40 mm applicator mounted (Zeiss, Oberkochen, Germany).

Wide local excision was performed, and the Intrabeam 600 system (Zeiss, Oberkochen, Germany) (**Figure 1**) delivered IORT sequentially. A 35-mm spherical applicator delivered 20 Gy to the surgical margin for 17 minutes. Intraoperative ultrasound determined that the applicator’s closest margin to the skin was approximately 1.3 cm, and the absorbed dose from TARGIT-IORT radiation on the skin’s surface was 1.96 Gy.

TARGIT-IORT and surgery were uneventful. The histology of the right breast revealed pathological stage IIA T2 N0 grade 3 IDC spanning 4.5cm x 3cm. The tumor was fully removed with clear margins. The tumor was ER/PR-positive but Her-2 negative. Adjuvant treatment with T-DM1 and radiation therapy would have been ideal, but due to the patient’s severe developmental delay, she couldn’t undergo infusions or daily radiation treatments. Therefore, it was determined that the aromatase inhibitor (A.I.), along with subcutaneous pertuzumab/trastuzumab/hyaluronidase, would be used for her adjuvant treatment. This treatment regimen has been ongoing for 10 months, and she is currently showing no evidence of disease during follow-up assessments (**Table 1**).

**Table 1:** Summary of patient data.

Patient	Age (Years)	Final Pathology	Pathological Size (mm)	Size of the Tumor Resected (cm)	Applicator Size (mm)	Laterality	Complications	Adjuvant Therapy
1	66	IDC	45	3.1 x 5.2 x 4.5	30	Right	Development Delay + Behavioral Disorder	Aromatase Inhibitor + Pertuzumab/Trastuzumab/Hyaluronidase
2	47	IDC + DCIS	25	5 x 5 x 2.4	35	Left	Intellectual Disability	Tamoxifen
3	70	DCIS	NA^*^	1.5 x 5 x 5.5	35	Left	Tardive dyskinesia	Aromatase Inhibitor + Trastuzumab
4	59	ILC	15	3.3 x 3 x 0.7	40	Right	Traumatic Brain Injury (TBI)	Aromatase Inhibitor

^*^No residual invasive carcinoma. Span could not be determined due to a few scattered foci.

### Patient 2

A 47-year-old female with intellectual disability presented after a diagnostic mammogram. She was unable to provide any history. However, her proxy mentioned that her mother had also been diagnosed with intellectual disability. Her ultrasound finding demonstrated left 11:00-12:00 o’clock 6 cm from the nipple a hypoechoic mass with irregular margins measuring 1.8 x 1.6 x 2.1 cm. Her right breast subareolar region showed a large, predominantly hypoechoic mass with mild lobulations measuring over 6 cm. An ultrasound-guided biopsy of the left breast revealed grade 2 moderately differentiated IDC. The tumor was diagnosed as clinical stage IA T1 N0 M0, and tumor markers were ER +, PR +, and Her-2 negative. Right breast biopsy revealed a fragmented fibroepithelial lesion, but the size of the lesion made phyllodes a differential. Her case was discussed at the MTB, and it was collectively decided to offer her a bilateral lumpectomy with left SLNB and left-sided TARGIT-IORT.

Wide local excision was performed on each breast, and TARGIT-IORT was delivered to the left breast using a 35-mm diameter spherical applicator, delivering a dose of 20 Gy for 20 minutes. The measured absorbed dose from the Intrabeam TARGIT-IORT system radiation on the skin surface was 1.01 Gy for the left breast.

The surgery and TARGIT-IORT were both uneventful. Histology of the left breast confirmed the presence of IDC measuring 2.5 cm with DCIS grade 3, ER/PR+, Her 2-, stage IIA T2 N0 M0. The tumor was fully removed with clear margins. The right breast was diagnosed as a benign phyllodes tumor spanning 7.4 cm with focal atypical lobular hyperplasia. The patient is 4 months postoperative and shows no signs of disease on follow-up; she is receiving tamoxifen as adjuvant therapy.

### Patient 3

A 70-year-old female with a history of schizophrenia managed with antipsychotic medication and subsequent tardive dyskinesia presented with a 7-month history of breast pain and a mass in her left breast. Subsequent mammography and ultrasound revealed a 2.2 x 1.1 x 1.5 cm mass at the left 8:00 axis, 3 cm from the nipple. Ultrasound-guided biopsy confirmed clinical stage IIA T2 N0 M0 left IDC, moderately differentiated, ER+, PR+, and Her-2 positive at the 8:00 axis. Surgical options were discussed, and she expressed a preference for lumpectomy. However, her tardive dyskinesia made her ineligible for external beam radiation.

The case was reviewed at the MTB, and it was decided collectively to offer her neoadjuvant treatment with Paclitaxel/Herceptin/Pertuzumab followed by lumpectomy and SLNB along with TARGIT-IORT.

Subsequently, wide local excision was performed, and TARGIT-IORT was delivered using a 35-mm diameter spherical applicator, delivering a dose of 20 Gy for 20 minutes. Intraoperative ultrasound determined that the closest margin of the applicator to the skin was approximately 11.2 mm, and the dose absorbed from TARGIT-IORT radiation on the skin surface was 2.67 Gy.

The surgery and TARGIT-IORT were uneventful. Histology of the left breast demonstrated a few foci of grade-3 DCIS with no residual invasive carcinoma following neoadjuvant chemotherapy. The span of DCIS could not be determined since there were only a few scattered foci. The tumor was stage 0 Tis N0 M0 with clear margins. Adjuvant treatment with Trastuzumab and an A.I. was initiated, and the patient showed no signs of disease on their 3-month postoperative follow-up.

### Patient 4

A 59-year-old female, an active smoker with a PMHx of traumatic brain injury (TBI), bipolar disease, depression, anxiety, COPD, and asthma, presented after an abnormal screening mammogram of the right breast. A subsequent diagnostic mammogram demonstrated a questioned mass in the right breast with indistinct margins, and due to the patient’s inability to tolerate further mammographic imaging, a targeted ultrasound was performed, identifying a mixed echotexture mass located at the11:00 o’clock axis 10 cm from the nipple. The biopsy of the right breast revealed invasive lobular carcinoma (ILC) ER+, PR negative, and Her-2 negative with clinical stage IA T1 N0 M0. Her PMHx of TBI resulted from a possible stroke that occurred 15 years ago secondary to a drug overdose, which resulted in involuntary movements and muscle stiffness along with a broad-based gait and increased lower extremity movements.

The case was reviewed at the MTB, and it was decided collectively to offer her lumpectomy with TARGIT-IORT. Wide local excision with right-sided TARGIT-IORT and SLNB was performed. A 40-mm applicator delivered 20 Gy in 25 minutes. Both surgery and TARGIT-IORT were uneventful. Intraoperative ultrasound determined that the closest margin of the applicator to the skin was approximately 8.5 mm, and the absorbed dose from TARGIT-IORT radiation on the skin’s surface was 4.28 Gy (**Table 2**). Histology of the right breast revealed grade 2 ILC forming a 1.5 cm mass. The tumor was stage IA T1 N0 M0, ER+, PR negative, and Her-2 negative and had clear margins. Adjuvant treatment with an A.I. was initiated, and she currently has no evidence of disease on her 6-month follow-up.

**Table 2:** Dose reported is for the closest skin bridge measurement (applicator to skin distance) as determined using ultrasound measurements localization measuring the 4 cardinal positions of superior, medial, inferior, lateral and has been determined using the validated model presented in Brodin et al. (82) The 95% confidence interval is shown in parentheses.

Patient	Treatment Time (min:sec)	Right Breast Closest Skin bridge distance (mm)	Dose to Skin Breast (Gy)
Patient 1	16:40	13.2	1.96 (1.73-2.24)
Patient 2	19:40	19.4	1.01 (0.93-1.12)
Patient 3	19:56	11.2	2.67 (2.36-3.03)
Patient 4	24:41	8.5	4.28 (3.85-4.77)

## Discussion

An intellectual disability is characterized by restrictions in intellectual functioning and adaptive behavior, encompassing practical, social, and conceptual skills. It typically manifests before the age of 22 years (34) with an Intelligence Quotient or IQ of at least 2 standard deviations below the mean (35). While the occurrence of breast cancer among women with ID is comparable to that in the general population (14–16), studies have revealed a significantly increased risk of overall and breast cancer-specific mortality in this group (17, 18). Furthermore, there is growing evidence suggesting that women with ID are more prone to a higher prevalence of risk factors associated with breast cancer, which places them at an increased risk of developing breast cancer when compared to their counterparts in the general population (36–38). These risk factors include nulliparity, inadequate physical activity, and elevated rates of obesity (36, 37, 39–42). Additionally, women with an ID exhibit limited knowledge regarding breast awareness and breast cancer (15), consequently resulting in late-stage presentation and poorer clinical outcomes. Similarly, psychiatric patients have breast cancer incidence that mirrors the general population but face higher mortality rates (19). Some of the contributing factors for higher mortality are that psychiatric patients have fewer breast cancer surgeries, receive less radiation therapy, and have more metastases at presentation than the general population (19). Additionally, psychiatric patients are impacted by nulliparity, obesity, and exposure to antipsychotics, which further elevates their risk of developing breast cancer (43–45). Multiple studies have shown that individuals with pre-existing disabilities are more likely to receive a mastectomy and less likely to receive chemotherapy and radiation therapy (46–49).

Amongst patients with ID and psychiatric illnesses, EBRT may not be feasible due difficulty staying still (DSS) during treatment. This could potentially result in either inadequate treatment or necessitate a more aggressive approach such as mastectomy. A major challenge in administering EBRT are patients who have DSS. Prior literature has demonstrated that performing EBRT can be challenging when patients can’t lie flat or appropriately abduct the arm (11, 12). In our study, each of the four patients faced obstacles that would have hindered their capacity to endure EBRT, stemming from physical discomfort or movement disorders that compromised their ability to remain still. In our case series, Patient 1 experienced challenges with arm abduction due to limited range of motion and could not even tolerate her biopsy procedure. Although Patient 2 was responsive to simple commands, there was uncertainty about her capacity to withstand EBRT. Conversely, Patients 3 and 4 exhibited movement disorders that rendered them unsuitable candidates for EBRT. Additionally, performing EBRT might have precluded the safe and/or consistent administration of radiation therapy in these patients. A study conducted by Sreeraman et al. (50) found that patients with pre-existing psychiatric conditions who were treated with radiation for head and neck cancer had a higher rate of treatment breaks than patients with no psychiatric history. Apart from this, healthcare providers may lean towards recommending radical surgical interventions for psychiatric patients due to issues related to patient cooperation (49). A study conducted by Abdullah et al. (51) found that patients with schizophrenia exhibited verbal or physical aggression toward their healthcare providers before radiation therapy was offered. Additionally, the overall financial burden in patients with pre-existing psychiatric illnesses undergoing EBRT is higher compared to non-psychiatric patients. Waddle et al. (52) conducted a study to assess the expenses associated with acute and follow-up care in psychiatric patients receiving radiation treatment. Their findings revealed that acute costs were significantly higher in the psychiatric group, with a difference of $3389 (95% confidence interval [CI] for difference, –$1993 to $8771; $45,293 vs $41,904; P = .039) (52). Moreover, follow-up costs were notably elevated in the psychiatric group, demonstrating a difference of $9653 (95% CI for difference, $1,642-$17,664; $28,084 vs $18,431; P = .003) (52).

To bridge some of these concerns, TARGIT-IORT can be a prudent option for patients where EBRT may not be feasible. TARGIT-IORT is a type of accelerated partial breast irradiation (APBI) that enables the delivery of a single high dose of radiation directly to the surgical margins shortly after tumor removal. It utilizes low-energy 50kVp photons to minimize scatter and radiation exposure to neighboring critical organs due to the steep dose fall-off past the applicator surface. For example, using a 30-mm applicator, the dose decreases by 49% at a distance of 5 mm from the applicator surface and by 28% at a distance of 10 mm (8, 53, 54). The advantage of utilizing TARGIT-IORT is that it allows for a single dose to be administered at the time of lumpectomy, which can be extremely beneficial for patients who can’t tolerate EBRT and/or have DSS. Patients who struggle with compliance and fail to complete their radiation treatment may otherwise be better suited for mastectomy (55). TARGIT-IORT offers these patients an alternative to mastectomy, thereby mitigating the increased morbidity and potential complications associated with this larger surgery. Furthermore, a major concern with administering EBRT is patients with DSS. A study by Kim et al. (26) highlighted the use of adjuvant radiation therapy in a patient with Huntington disease with choreiform movements. Their challenge was to control these choreiform movements sufficiently enough to provide EBRT, which they achieved with olanzapine; however, this led to treatment delay. Conversely, TARGIT-IORT, performed under anesthesia, circumvents issues related to involuntary movements, making it a preferable option and avoiding the additional steps and risks associated with the management of movement disorder during EBRT and further treatment delays.

In addition to these special considerations, the utilization of TARGIT-IORT provides further benefits compared to EBRT. The use of WBI has been associated with various adverse effects, notably increased non-breast cancer-related mortality (56). WBI also increases the risk of secondary cancers and heart disease (57–59). In a study involving 134 breast cancer patients, 90 of whom underwent WBI, the rate ratio for lung cancer incidence over ≥ 10 years was 2.10 (95% CI, 1.48 to 2.98; P = 0.001) (57). Additionally, WBI has been linked to various heart diseases, including ischemic heart disease, myocardial infarction, valvular disease, coronary stenosis, pericarditis, and other cardiac abnormalities (57–59). WBI can also exacerbate cosmetic outcomes due to skin toxicity and fibrosis, especially when boosting the tumor bed (60). In contrast, TARGIT-IORT significantly reduces the non-breast cancer-related mortality rate (45 vs. 74 events for TARGIT-IORT and EBRT, respectively; hazard ratio 0.59; 95% CI, 0.40 to 0.86; P=0.005), including cardiovascular causes (56). Additionally, a randomized trial involving 2,298 patients conducted by the TARGIT group found that IORT was non-inferior to EBRT, with local recurrence rates of 2.11% for IORT compared to 0.95% for EBRT (56). Moreover, the same group analyzed long-term outcomes in these patients, assessing tumor size, grade, receptor status, and lymph node status that affected local recurrence-free survival, as well as the impact of local recurrence on distant relapse and mortality (61). They observed no difference in 5-year local recurrence-free survival between TARGIT-IORT and EBRT across all tumor subgroups. An additional benefit of TARGIT-IORT is that it may reduce the risk of secondary lung cancers, which are commonly associated with smokers undergoing EBRT (62). Notably, neither Patient 3 nor Patient 4 in our study, whether former or active smokers, experienced complications during their respective follow-up periods. The TARGIT-A trial randomized 3451 patients to WBI (1730) or TARGIT-IORT (1721) to analyze toxicities and complications. Wound-related complications were similar between the two groups, but TARGIT-IORT had significantly fewer grade 3 or 4 toxicities and better cosmesis than WBI (63, 64). TARGIT-IORT has also been shown to yield better breast-related quality of life and overall quality of life (65, 66). Moreover, because of its shorter treatment duration and fewer visits, TARGIT-IORT may result in higher patient compliance, potentially improving the overall patient experience (67). This is of particular significance for individuals with ID residing in nursing homes, who may otherwise require frequent visits to complete their radiation cycles, as well as for psychiatric patients who may be prone to noncompliance with their radiation treatments. Thus, TARGIT-IORT becomes a more feasible alternative for these patients.

When administering radiation therapy, another crucial factor to consider is pain, particularly in patients with ID. There is a paucity of research on pain in individuals with ID, possibly because they are routinely excluded from pain studies. This exclusion could be attributed to the numerous functional limitations and underlying neurological conditions, which often complicate pain presentation and measurement (68). Additionally, long-standing beliefs about pain insensitivity or indifference in ID patients may further contribute to this gap in research (69). However, emerging evidence suggests that individuals with ID may be more sensitive to painful stimuli under certain circumstances, contrary to previous beliefs (70, 71). They may exhibit greater pain-evoked potentials (72–74) and are more likely to experience chronic pain compared to typically developing peers (75). Estimates indicate that chronic pain prevalence in ID averages around 70%, considerably higher than the general population (76, 77). Upon comparing TARGIT-IORT vs. EBRT in terms of pain, Andersen et al. (78) conducted a study revealing that persistent pain in the breast area, side of the chest, axilla, or arm after EBRT was reported in 33.9% of cases, compared to 24.6% in the TARGIT-IORT group (P = 0.11). Similarly, Corcia et al. and Welzel et al. found that EBRT patients experienced moderately higher levels of breast and arm pain compared to TARGIT-IORT. This finding is particularly relevant for Patients 1 and 2 in our study, who both had ID and were non-verbal. Receiving EBRT may have resulted in higher levels of persistent pain for them, which they would been unable to express. Additionally, elderly patients, including Patients 1 and 3 in our study, might have potentially benefited from surgery and endocrine therapy alone, avoiding radiation treatment altogether (79). However, the decision to administer radiotherapy to these patients was influenced by the findings of the Cancer and Leukemia Group B (CALGB) 9343 trial, which demonstrated that combining radiation therapy with endocrine therapy improved locoregional recurrence prevention in women aged 70 and above (80). Moreover, the PRIME II study, a randomized trial involving 1,326 patients with non-metastatic hormone receptor-positive breast cancer, all aged 65 and older, who underwent breast-conserving surgery and were receiving adjuvant hormone therapy, found a significantly higher rate of local recurrence after 10 years in patients who did not receive radiation therapy compared to those who did (9.8% vs. 0.9%) (81), thereby supporting our decision to include radiotherapy in our patient’s treatment plan. Furthermore, it is noteworthy that none of the 4 patients experienced any acute or chronic toxicities following breast-conserving surgery and TARGIT-IORT. In addition, all of the patients in our study tolerated the TARGIT-IORT well and developed no local recurrence on follow-up. Notably, all four individuals received outpatient treatment, avoiding potential complications associated with hospitalization. As the number of breast cancer cases increases, there may be a higher probability of encountering patients with DSS; thus, future studies are required to further evaluate the utility of TARGIT-IORT vs. WBI for patients with DSS in order to establish new guidelines.

Our study has several limitations. First, our study has a small sample size. Second, the retrospective nature of the study introduces inherent limitations. Third, our study had a short follow-up period. Finally, our study lacked measurement of pain scale, a cosmesis scale, and patient-reported outcomes, which could have provided a more comprehensive understanding of the treatment effects in this specific group of patients.

## Conclusion

Our study underscores the potential use of TARGIT-IORT as a viable treatment option for breast cancer patients with intellectual and psychiatric disabilities. Unlike traditional EBRT, TARGIT-IORT offers a single radiation dose, addressing challenges associated with compliance or DSS. Our findings demonstrate positive outcomes and tolerance, especially in patients where standard oncologic procedures are difficult to achieve. TARGIT-IORT could also benefit breast cancer patients with concurrent movement disorders like Parkinson’s disease and other movement disorders. Nonetheless, future studies are needed to reinforce its applicability for patients with DSS.

## Data Availability

The raw data supporting the conclusions of this article will be made available by the authors, without undue reservation.
